# The missing link in the blue economy: Empowering the next generation through blue skills and ocean literacy

**DOI:** 10.1017/cft.2026.10042

**Published:** 2026-07-07

**Authors:** João Canning-Clode, Diane Esson, João Gama Monteiro, Ana Dinis, Carlos Andrade, Filipe Alves, Sonia K.M. Gueroun, Dinarte Vieira, Patricia Nunes, Barkha Mossae, Arthur Tuda, Imogen E. Napper, A. Meriwether W. Wilson, Alexander W. Tudhope

**Affiliations:** 1 MARE - Marine and Environmental Sciences Centre/ARNET - Aquatic Research Network, Agência Regional Para o Desenvolvimento da Investigação Tecnologia e Inovação (ARDITI), Funchal, Madeira, Portugal; 2 Faculty of Life Sciences, University of Madeira, Funchal, Madeira, Portugal; 3 Smithsonian Environmental Research Center, Edgewater, MD, USA; 4 IUCN - International Union for Conservation of Nature, Eastern and Southern Africa Office (ESARO); 5 WIOMSA - Western Indian Ocean Marine Science Association, Zanzibar, Tanzania; 6 School of Biological and Marine Sciences, University of Plymouth, Plymouth, UK; 7 School of GeoSciences, University of Edinburgh, Edinburgh, UK

**Keywords:** blue economy, ocean sustainability, environmental stewardship, youth engagement, inclusive innovation

## Abstract

The rapid expansion of the blue economy sector, now employing millions worldwide, reveals a growing gap between technological ambition and human capacity. Despite the sector’s promise, ocean literacy remains critically low, and young people often lack pathways to participate in sustainable ocean careers. Addressing this gap requires investment in accessible, interdisciplinary training to develop the blue skills essential for future ocean sustainability. Here we highlight the urgent need to invest in *blue skills* and foster a new generation of interdisciplinary, creative and socially engaged ocean professionals. Using an island in the Atlantic Ocean (Madeira) as a model environment, we introduce the emerging concept of a *Blue Skills Academy*, a cost-effective and inclusive educational programme designed to empower youth through experiential learning that bridges ocean science, several fields of engineering, social sciences, arts and leadership skills. We outline how the *Blue Skills* Academy programmes can form the foundation of *Blue Campuses*; physical locations where education, research, innovation, entrepreneurship and community engagement converge around ocean-based opportunities. Key components of *Blue Campuses* include (1) academies that facilitate ocean-positive skills training and (2) physical spaces that foster the exchange of ideas across blue economy disciplines. Combined, the *Blue Skills Academy* and *Blue Campuses* can lead the way in ensuring sustainable ocean interactions are not merely an international policy ambition but something that any member of society can fully participate in. We believe investing in people and connections, more than just technology and businesses, is the missing link to achieving an equitable and responsible blue economy.

## Graphical abstract

**Figure:**
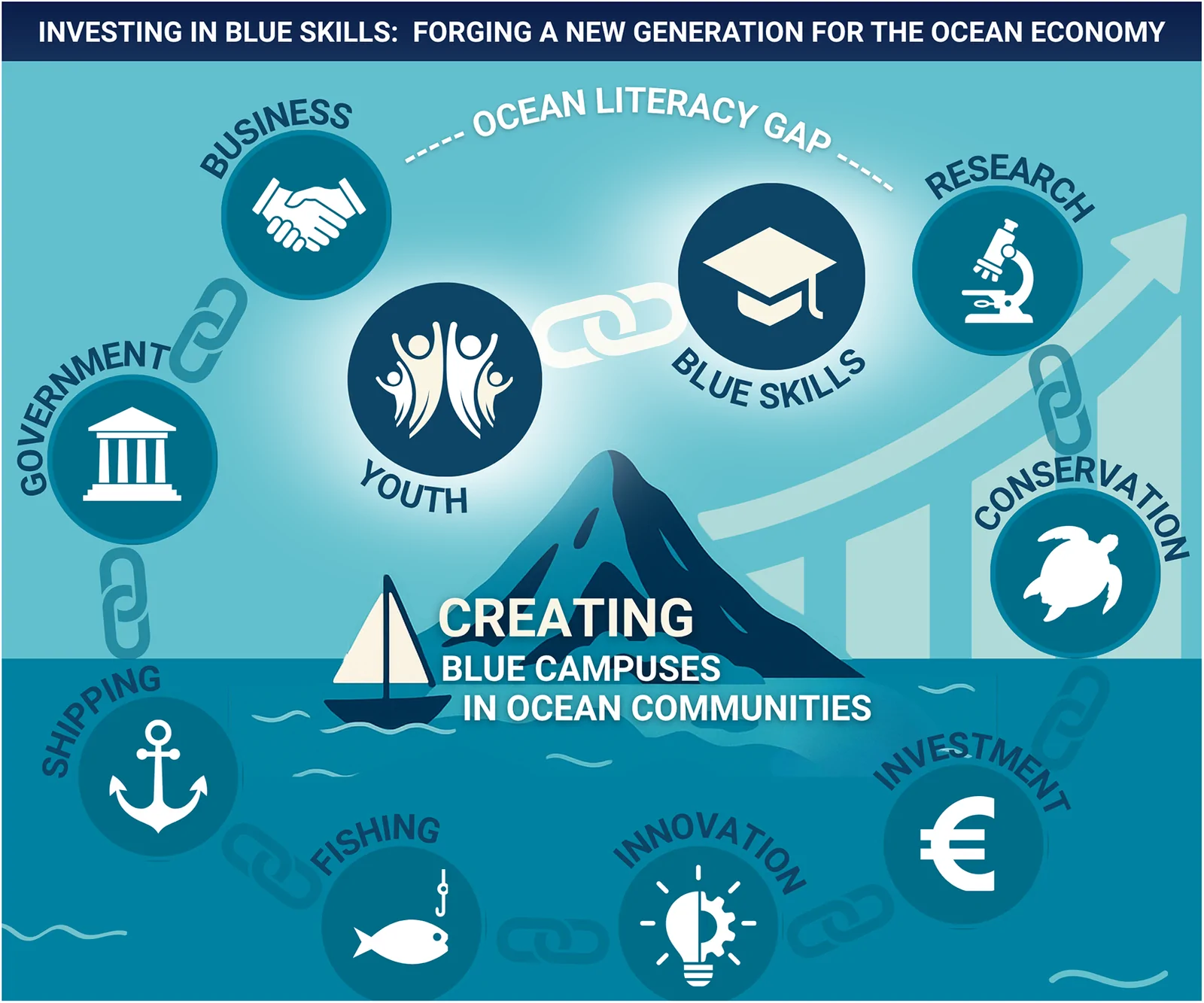

## Resumo

A rápida expansão do setor da economia azul, que atualmente emprega milhões de pessoas em todo o mundo, revela um fosso crescente entre a ambição tecnológica e a capacidade humana. Apesar do seu potencial, a literacia do oceano permanece criticamente baixa, e muitos jovens continuam sem vias de acesso a carreiras ligadas a um oceano sustentável. Colmatar esta lacuna exige investimento em formação acessível e interdisciplinar que desenvolva as competências azuis essenciais para a sustentabilidade oceânica do futuro. Neste artigo, destacamos a necessidade urgente de investir em competências azuis e de formar uma nova geração de profissionais do oceano que sejam interdisciplinares, criativos e socialmente envolvidos. Utilizando uma ilha no Oceano Atlântico (Madeira) como modelo, apresentamos o novo conceito de Academia Azul, um programa educativo inclusivo e de baixo custo, concebido para capacitar jovens através de uma aprendizagem prática, integrando ciências do mar, vários campos da engenharia, ciências sociais, artes e ainda competências de liderança. Descrevemos ainda como programas deste tipo podem constituir a base para um Campus Azul – um espaço físico onde formação, investigação, inovação, empreendedorismo e envolvimento comunitário convergem em torno de oportunidades ligadas ao oceano. Em conjunto, estes dois conceitos, academias que promovem competências orientadas para um oceano saudável e campus que fomentam a troca de ideias entre disciplinas da economia azul, podem assegurar que interações sustentáveis com o oceano deixem de ser apenas uma ambição política internacional e se tornem algo ao alcance de qualquer pessoa. Acreditamos que investir em pessoas e em conexões, mais do que apenas em tecnologia ou no setor económico, é o elo que falta para atingirmos uma economia azul equitativa e responsável.

## Impact statement

The future of the ocean will be shaped not only by science and policy, but also by those who have the access and ability to engage with the ocean, now and in the future. Today, millions of young people living beside the sea grow up without ever imagining themselves in ocean careers. This *Perspective* introduces a new vision to change that: the *Blue Skills Academy* – a model for vocational training that gives young people the tools, confidence and community to engage with the ocean in a positive way. Building on this foundation, we also propose a global network of *Blue Campuses*, physical spaces where learning, innovation, leadership and community engagement intersect. By connecting these campuses through capacity sharing and interdisciplinary learning on a global scale, this model has the potential to transform access to the blue economy and expand who can contribute to ocean solutions. *Blue Skills Academies* and *Blue Campuses* can expand access to skills, networks and creative spaces, and in turn cultivate the next generation of ocean professionals.

## Introduction

The blue economy encompasses all ocean-based enterprises, including fisheries, tourism, aquaculture, renewable energy, biotechnology and shipping, and has become one of the world’s fastest growing economic sectors (Lee et al., 2020; European Commission, 2025). In the European Union alone, blue economy activities employ ~4.9 million people and contribute €250 billion in gross value added, with continued expansion across all sub-sectors (European Commission, 2025). Comparable growth trajectories are emerging globally, particularly across small islands and large ocean-developing states, where ocean resources play a central role in livelihoods and economic resilience (Wenhai et al., 2019; Cisneros-Montemayor et al., 2021).

However, this expansion exposes a widening ‘blue skills gap’, a shortfall of trained professionals, technicians, educators and leaders needed to support sustainable and equitable ocean development (Bennett et al., 2019; Martínez-Vázquez et al., 2021). Many coastal and island regions lack access to affordable training, research infrastructure and clear career pathways, resulting in the systematic exclusion of local youth and communities from emerging opportunities in ocean-related sectors. Increasing tensions between ocean industries (e.g. tourism vs. fisheries; offshore energy vs. conservation) underscore the growing need for integrated, cross-sector training and collaboration to reduce conflict and promote shared stewardship (Martínez-Vázquez et al., 2021). This ‘blue skills gap’ is not born of a single problem but multi-dimensional challenges. It reflects an absence of accessible training opportunities in island and coastal regions, a fragmentation of efforts across disciplines, sectors and institutions (Andrews et al., 2020), and the lack of a shared definition of what *blue skills* are and how they should be recognised (Martínez-Vázquez et al., 2021). Distinguishing between these challenges is relevant because each requires a different solution, and any substantial response should tackle all three simultaneously.

These challenges are further intensified by a widespread lack of ocean literacy. A recent survey shows that 50% of young people do not understand how the ocean influences their lives, nor how their actions affect ocean health (Back to Blue, 2023). Early ocean education models focused on transferring knowledge, yet evidence shows that knowledge alone rarely drives sustained pro-ocean behaviour (McKinley et al., 2023). Contemporary frameworks emphasise participation, emotional connection and the inclusion of diverse knowledge systems, including indigenous and local perspectives, to foster meaningful and lasting engagement (Harden-Davies et al., 2022; McKinley et al., 2023; UNESCO IOC, 2024; Hoareau, 2025). Effective engagement relies not only on understanding the ocean scientifically, but also on feeling connected to it personally, culturally and emotionally. This is where the arts and social sciences can provide valuable and often overlooked pathways to ocean literacy. Through storytelling, creative expression and social inquiry, they help translate scientific knowledge into lived experience, creating meaning, empathy and a sense of belonging, elements consistently associated with pro-environmental action and civic participation (Loroño-Leturiondo and Olazabal, 2025). Rather than replacing science, these disciplines complement and expand it, inviting broader participation in ocean issues from individuals who might not otherwise see themselves reflected in marine science or blue economy careers.

Addressing our global blue skills and ocean literacy gaps is not only essential for socio-economic development and more sustainable connections with the ocean, but also realising our collective vision of a sustainable future. Achieving the UN Sustainable Development Goals 4 (Quality Education), 8 (Decent Work), 13 (Climate Action) and 14 (Life Below Water), and fulfilling the ambitions of the UN Ocean Decade, depend on promoting human capacity in parallel with scientific and industrial growth (Cisneros-Montemayor et al., 2021). Embedding sustainability into blue economy strategies requires a deep understanding of local knowledge systems and leadership from people with a strong lived connection to marine environments within their day-to-day life (Hoareau, 2025). Recent global policy frameworks reinforce this priority: the new UN Agreement on Marine Biological Diversity of Areas Beyond National Jurisdiction (ABNJ) calls for capacity building and transfer of marine technology to ensure equitable access to and stewardship of ocean resources (BBNJ Treaty, 2023). A sustainable blue economy cannot be achieved through infrastructure and technology alone; it must also invest in the human capacities, identities and aspirations that shape how communities engage with the ocean.

In this context, this *Perspective* aims to address one of the most overlooked barriers in the global ocean agenda: the lack of accessible *blue skills* training opportunities that keep pace with the rapid expansion of the blue economy. In response, we propose a conceptual framework for building *blue skills* that places people, particularly youth, at the centre of ocean sustainability. This human-driven approach complements technological and policy innovation by ensuring that investments also strengthen training, education and capacity sharing. Using Madeira’s emerging *Blue Skills Academy* as a pilot initiative, we illustrate how small islands can act as living laboratories for ocean literacy and ocean skills development by combining hands-on practice, community engagement and low-cost, open-source tools for ocean exploration, monitoring and conservation. We further explore the concept of international capacity-sharing networks, in which expertise, methods and affordable technologies circulate across borders, particularly between small islands and developing coastal regions. This model embraces transdisciplinarity, integrating natural sciences, engineering, social sciences and the arts as equally vital components of ocean education. In doing so, we hope to forge the missing link in inspiring ocean stewardship and preparing the next generation to pursue meaningful ocean-positive careers.

Throughout this *Perspective*, we use the terms ‘interdisciplinary’ and ‘transdisciplinary’ in specific and complementary ways. Interdisciplinarity refers to the integration of knowledge, methods and perspectives from multiple academic disciplines to address a shared problem (Kelly et al., 2019; Blythe and Cvitanovic, 2020). Transdisciplinarity goes further, encompassing collaboration and co-creation of knowledge not only across disciplines but also with non-academic actors including communities, practitioners, policymakers or artists, an approach increasingly recognised as essential for supporting early career researchers in interdisciplinary fields (Kelly et al., 2019; Kadykalo et al., 2026). While the *Blue Skills Academy* is grounded in interdisciplinary learning, its deeper ambition is broader and transdisciplinary: to democratise knowledge production, legitimise diverse ways of learning, including local and indigenous perspectives, and to engage those who have historically been excluded from ocean-related education and careers (Box 1).

Box 1. Key blue concepts

To clarify the terminology used throughout this *Perspective* essay, we summarise next the key ‘Blue’ concepts underpinning this work.

Sustainable and equitable blue economy – The sustainable and equitable use of ocean resources for economic growth, improved livelihoods and jobs while maintaining the health of marine ecosystems. It includes activities such as fisheries, tourism, maritime transport, renewable energy and biotechnology, and seeks to balance economic opportunity with environmental protection and social equity (World Bank and UNDESA, 2017; Wenhai et al., 2019; Bennett et al., 2019; Lee et al., 2020).

Blue skills – The combination of practical, technical, social and creative aptitudes that enable people to contribute to a sustainable blue economy. These skills span marine science, conservation, engineering, communication, entrepreneurship, leadership and the arts, promoting inclusivity, innovation and accessibility across ocean sectors.

Blue skills academy – An educational programme designed to cultivate *blue skills* through experiential and interdisciplinary learning. Grounded in ocean science and conservation and strengthened through cross-disciplinary partnerships including aspects of finance and business, the Academy provides guidance, inspiration and hands-on practice in pursuing ocean-positive careers. With programmes spanning workshops to gap year-style courses, the *Blue Skills Academy* provides accessible opportunities to explore and aid employability within the blue economy to youth and life-long learners alike.

Blue campus – A physical and symbolic space where ocean-focused education, research and innovation intersect. It serves as a local hub that brings together scientists, educators, entrepreneurs, artists and communities to co-create solutions and foster ocean stewardship.

Blue campus ecosystem – A dynamic, global network of *Blue Campuses* across the world connected by shared values and exchanges. Each campus adapts to local needs while remaining linked to others through training programmes, workshops and digital collaboration, forming a living system of reciprocal learning and innovation.

Blue futures – The overarching vision of sustainable, equitable and ocean-positive societies. Building blue futures means empowering people, especially younger generations, to transform ocean knowledge into action and leadership, bridging education, economy and community for long-term planetary well-being.

## Concept spotlight: The *Blue Skills Academy* in Madeira

Madeira, an oceanic island in the Eastern North Atlantic, is shaped by a long maritime history that includes artisanal fishing, transatlantic shipping and ocean-centred tourism (García, 2017; Castro et al., 2020). Despite this strong maritime identity, formal ocean-related education and blue economy career pathways remain limited for young people. This combination of deep cultural connection to the sea and restricted access to ocean opportunities makes Madeira an ideal *living laboratory* to explore new approaches to ocean capacity-sharing and human-centred *blue skills* development.

Youth in Madeira grow up surrounded by the sea yet many remain disconnected from maritime- and marine-related careers (e.g. ocean science, maritime engineering, conservation) and from the emerging blue economy sector (e.g. aquaculture, sustainable fisheries, eco-tourism). The *Blue Skills Academy* emerges, therefore, as a response to this gap. It represents a forward-looking vision designed to transform proximity into opportunity by linking ocean literacy, conservation, science, arts, creativity and employability. Although still in its conceptual phase, early design steps, partnerships and modest sponsorships have enabled the implementation of preliminary workshops that lay the groundwork for broader implementation. The idea is currently being shaped within MARE-Madeira, the regional research unit of the Marine and Environmental Sciences Centre in Portugal, which provides the scientific, logistical and community foundation to bring the concept to life (MARE-Madeira, 2025). Unlike formal academic institutions, it is designed to operate outside traditional academic structures, prioritising accessibility and inclusion for youth and communities currently excluded from ocean-related education and careers. Notably, within traditional structures, there are numerous initiatives for inspiring young people with ocean-related education and careers, such as the Blue Schools, SEA BEYOND or Blue Curriculum programmes for school children (UNESCO-IOC, 2026) or dedicated Masters programmes that connect ocean science, conservation and social impact for advanced education, such as the Master’s in Marine Living Resources at NOVA Lisbon University, Portugal or the Master’s MARRES programme at Université Côte d’Azur, France. Yet, there remains a gap in helping those youth inspired by the ocean yet unprepared to choose between innumerable blue economy opportunities and ocean-related careers, be it academic, vocational, entrepreneurial or creative. As such, and rather than replacing traditional education models, the *Blue Skills Academy* is conceived as a complementary layer, one that connects existing formal pathways to informal experiential learning and career exploration.

The *Blue Skills Academy* is guided by five core principles that underpin both its philosophy and design (Figure 1). First, the *Academy* is rooted in accessibility and inclusion, offering informal and open learning pathways outside traditional academic structures such that young people from diverse educational and socio-economic backgrounds can participate. For example, participation in the *Academy’s* pilot workshops in 2024 and 2025 was free of charge, advertised in accessible community spaces and drew 20 to 30 participants each from five municipalities across Madeira Island, spanning diverse educational and socio-economic backgrounds and achieving gender balance. Second, the *Academy* prioritises sustainable and open-source innovation, using low-cost tools, open-source hardware and software and re-purposed technologies to democratise ocean exploration and ensure scalability beyond well-funded institutions. Looking ahead, *Academy* modules on innovation and engineering will challenge participants to build and deploy low-cost ocean monitoring instruments using open-source platforms such as Arduino and Raspberry Pi, and tools like OpenCTD and OpenROV, enabling skills that can be replicated without institutional budgets or proprietary equipment. This vision is grounded in ongoing low-cost innovation already being developed at MARE-Madeira and beyond, including novel instrumentation for deep-sea research (Radeta et al., 2025; Esson et al., 2026). Third, the *Academy* is inherently transdisciplinary, integrating natural sciences, engineering, social sciences and the arts to promote systems thinking and creativity. Learning experiences will be co-designed with local fishers, artists, entrepreneurs and community representatives alongside scientists and engineers, ensuring that knowledge flows in multiple directions rather than solely from *Academy* to participant. Fourth, the *Academy* emphasises youth empowerment. With a focus on teenagers and young adults, thereby aiding final decisions on school subjects and post-school career pathways the *Academy* enables participants to lead their own ideas and initiatives, build confidence and agency and gain practical, transferable skills for ocean-positive futures. In future leadership modules, for instance, participants will develop and pitch their own ocean-positive business ideas, receive mentorship from professionals and build practical skills and confidence about realising their own ocean-related careers. Finally, the *Academy* is anchored in community engagement, connecting schools, NGOs, research institutes and local enterprises to strengthen Madeira’s blue identity and promote collaboration across ocean sectors. This principle will be enacted through formal partnerships across the island and through public-facing events that invite the broader community to engage with *Academy* activities, outputs and participants.

**Figure 1.**
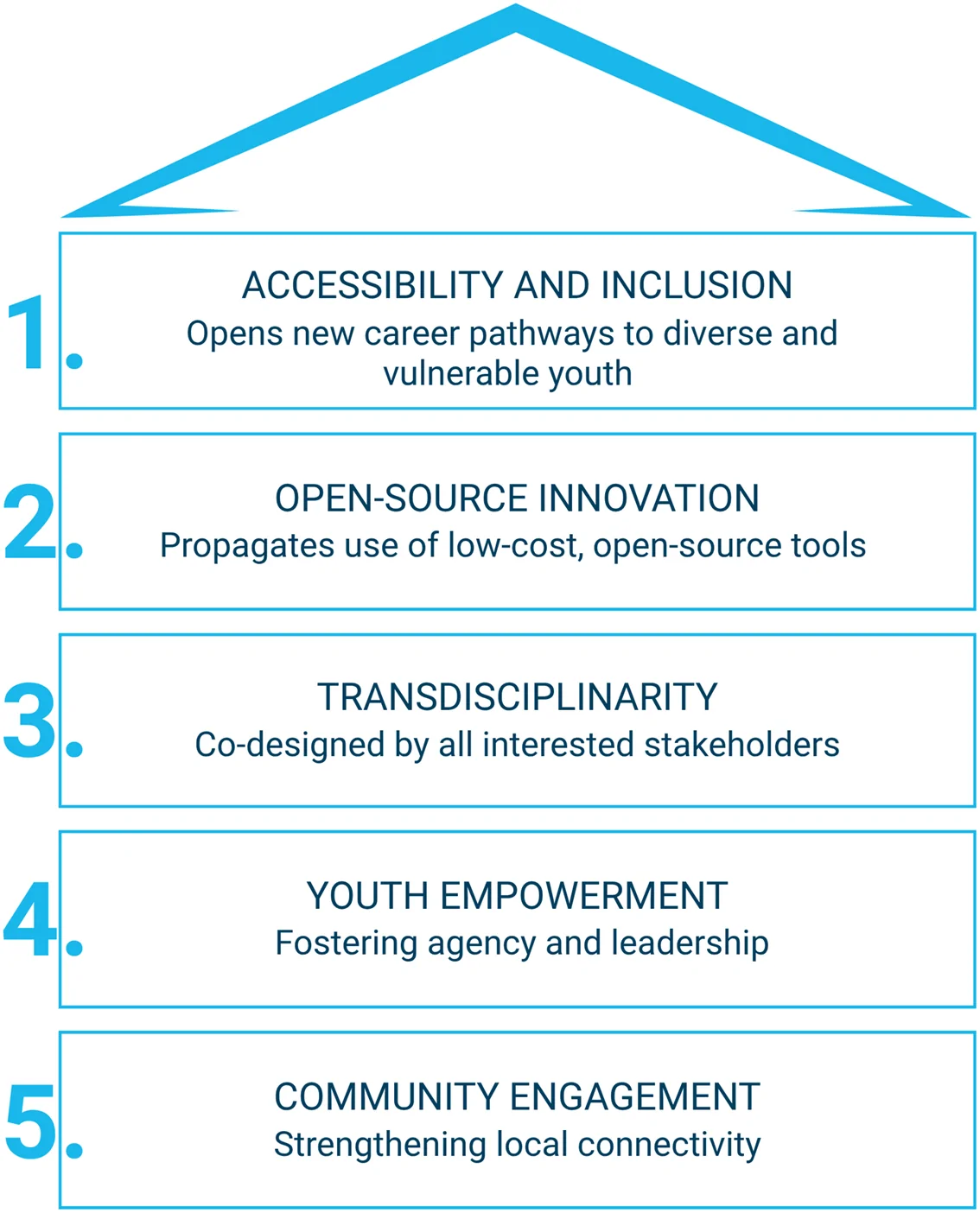
The five core principles guiding the *Blue Skills Academy*’s philosophy and design. Figure 1. long description.

The vision of a *Blue Skills Academy* builds on, but is distinct from, existing models of interdisciplinary marine education and research. Well-established institutions such as the Fisheries and Marine Institute of Memorial University of Newfoundland (one of Canada’s most comprehensive centres for ocean education, training and applied research), as well as the Centre for Marine Socioecology in Australia, which integrates disciplines across natural and social sciences to address complex marine sustainability challenges (Blythe and Cvitanovic, 2020), demonstrate the transformative potential of place-based, interdisciplinary ocean institutions. The *Blue Skills Academy* concept draws inspiration from these models while addressing areas of development that they do not fully support. Specifically, by operating outside formal academic structures, the *Blue Skills Academy* offers a low-risk, low-cost entry point for youth and community members interested in exploring these topics more freely and locally before committing to a multi-year programme abroad. Furthermore, the *Blue Skills Academy* expands the possibilities of ocean career exploration by including arts, entrepreneurship and creative practice alongside more traditional science and policy pathways, giving equal weight to a broad spectrum of ocean-positive opportunities.

Since 2024, two pilot workshops have already been implemented in Madeira within this *Blue Skills Academy* framework. Designed as modules that can fit within the broader *Academy* programme, these workshops (the Blue Startups Workshop and the Blue Stories Workshop) focused on the ‘blue skills’ of entrepreneurship and communication respectively. With total attendance of 50 young people aged 16–30 from five municipalities, including graduates who are un- and under-employed from a range of backgrounds and regions of the island, these workshops connected participants to experienced professionals from a range of ocean careers within each workshop theme and provided hands-on learning opportunities to build understanding of and confidence in pursuing such careers.

These pilot workshops and the *Blue Skills Academy* focus on empowering youth through inclusive, experiential ocean-skills training. Moreover, their full transformative potential emerges when such programmes are embedded within broader physical and social infrastructures: a vision we refer to as the *Blue Campus.*

## Vision – Part 1: Blueprint for a *Blue Campus*

The long-term vision emerging from the *Blue Skills Academy* concept is the creation of a *Blue Campus*: a place and infrastructure that can support the academy, where science, arts, innovation, education, entrepreneurship, policy, responsible recreation and creativity converge to shape sustainable ocean futures (Figure 2). While the *Academy* focuses on training and skills development, the *Campus* represents the surrounding environment, a physical and intellectual space that enables ideas to grow, collaborations to take shape and solutions to be tested.

**Figure 2.**
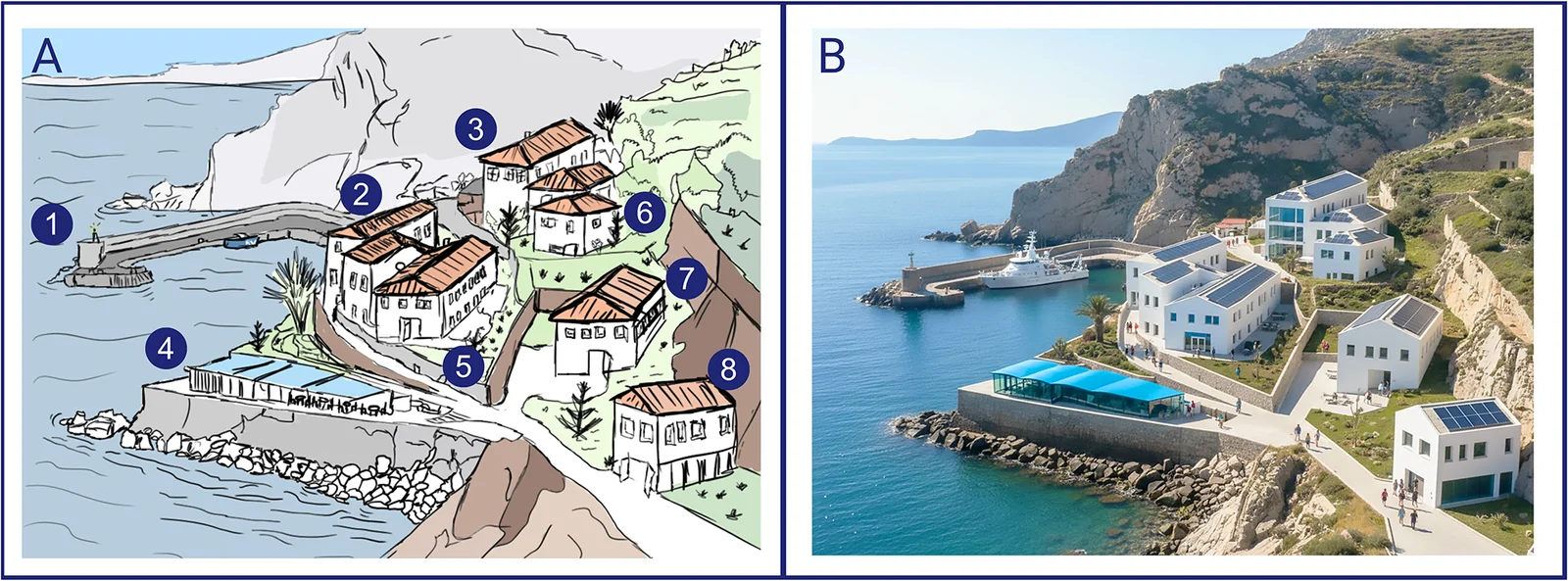
*Blue Campus* concept: A – Sketch of idealised campus space and facilities including (1) harbour for research vessels and responsible recreation, (2) *Blue Skills Academy* and marine science centre, (3) *Exploraquarium* for hands-on and virtual reality ocean science exhibits, art exhibitions and community meeting spaces, (4) cafe and outdoor space, (5) offices for startups and innovation workshops, (6) conservation departments and other government offices, (7) residence hall for visiting ocean leaders, scientists, academy students and artists and (8) recreational centre. B – AI interpretation of campus sketch performed in *Adobe Firefly.* Figure 2. long description.

In Madeira, we are working to secure a location and designated infrastructure to support such a campus. The concept design already involves a broad constellation of partners, including regional government, small businesses, NGOs, schools, youth organisations and international collaborators. Through this cooperative model, the *Campus* is conceived not as a top-down infrastructure project but as a co-created environment that responds to community needs; from better access to information and training, to job creation and entrepreneurship, to artistic and scientific exchange.

Early proof of concept for this approach has already begun to emerge. The ‘Impossible Things Workshop’, organised in Madeira during 2025 under the Horizon Europe project *TWILIGHTED* and endorsed by the UN Ocean Decade, brought together 40 participants from 12 countries and four continents, ranging from artists to entrepreneurs, from high-school students to retirees, to reimagine deep-sea research and exploration (Esson et al., 2025, 2026). The workshop demonstrated the transformative power of intergenerational and cross-sector collaboration: one young participant from the private sector decided to pursue a master’s degree in ocean sustainability after the event and another is now actively using his background in artificial intelligence to aid ocean research, showing how exposure to new professional and creative networks can shift aspirations and open life-changing opportunities.

Historic models demonstrate the power of an ecosystem approach to inspire new connections, collaborations and innovations. Many reference the close connectedness of academia, industry and finance in Silicon Valley as a key enabler to the area’s success in leading the digital revolution since the 1980s (Saxenian, 1996; Kenney, 2000). Similarly, the proximity of artists across a wide range of disciplines in 1400s Florence, which created opportunities for mentorship, competition and daily exposure to new techniques, is said to be responsible for the explosion of artistic talent that emerged from the city in the late fifteenth and early sixteenth centuries (Coyle, 2010). The blue economy lacks a place-based ecosystem where its multi-varied participants can regularly interact in constructive and creative ways. The *Blue Campus* seeks to provide such a space.

In this way, the *Blue Campus* is imagined as more than a collection of facilities; it is a living system of exchange that bridges disciplines, generations and cultures. It provides a physical and accessible space where diverse perspectives cross paths to generate new ideas for ocean sustainability. Crucially, the ideas generated within the *Blue Campus* flow back into the *Blue Skills Academy*, enriching training, stimulating innovation and creating practical employment pathways for the next generation.

## Vision – Part 2: Scaling through exchange

The *Blue Campus* concept is intentionally designed for adaptation and ‘franchising’ – not in a commercial sense, but in the sense of a model that can be reproduced and locally adapted in multiple places. While remaining connected through shared values of affordability, inclusion and interdisciplinarity, each *Blue Campus* should evolve according to its own cultural identity, ecological realities, local or indigenous knowledge systems and socio-economic context. In this way, Madeira’s *Blue Campus* is not a template to export, but a proof of concept demonstrating how island and coastal communities can build their own centres of ocean learning that are locally grounded yet globally connected.

Over time, this model could give rise to a global *Blue Campus* network (Figure 3): autonomous yet interconnected hubs linked through shared learning programmes, workshops and digital exchanges. Each campus would contribute to a global learning continuum, strengthening ocean literacy, innovation capacity and inclusion. The potential international scope of this model, connecting many campuses through exchanges in innovation, art, education, internships and student mobility, places Madeira as one of many autonomous hubs within a wider *Blue Campus* ecosystem.

**Figure 3.**
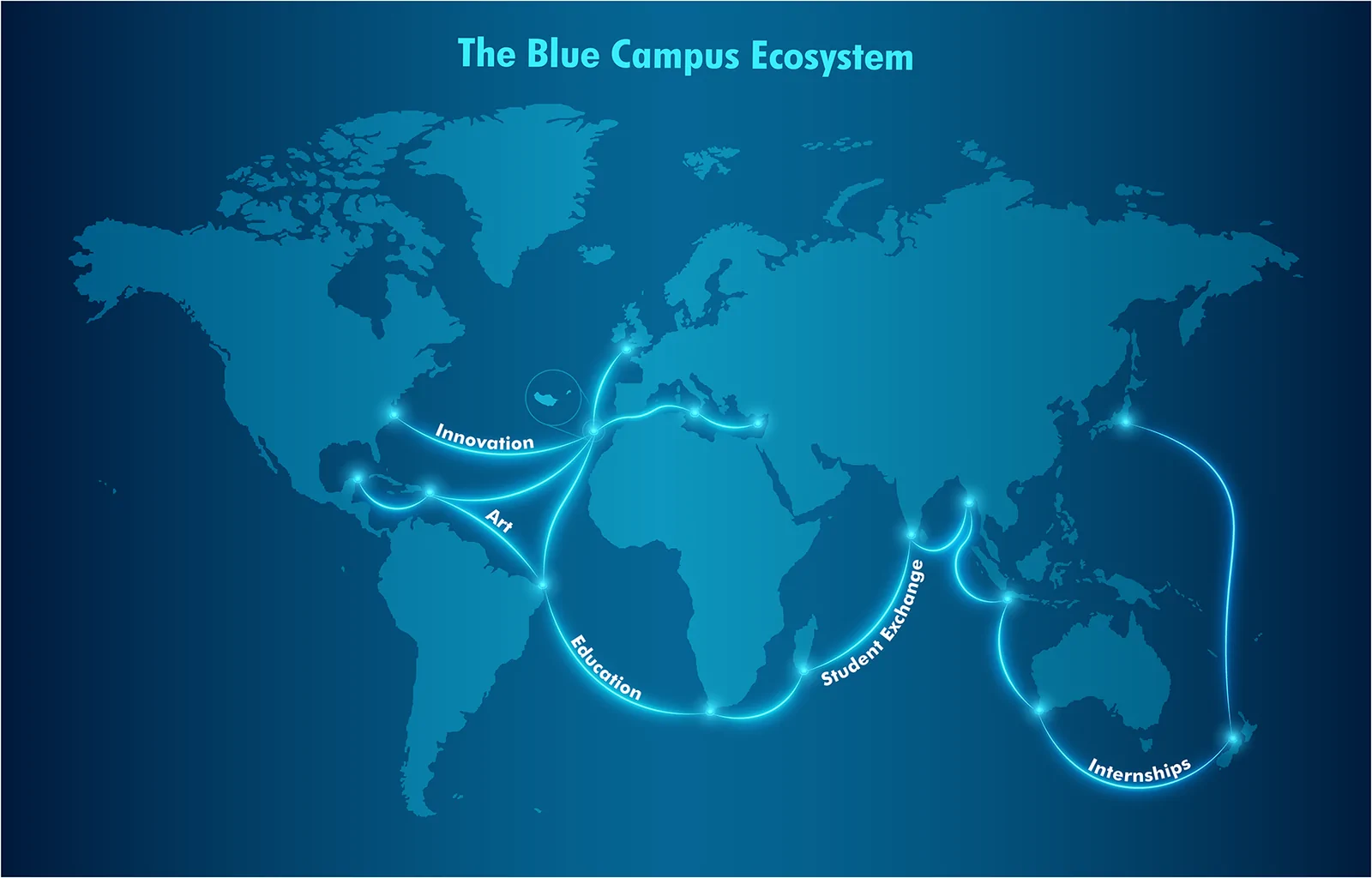
The *Blue Campus* ecosystem: Conceptual map illustrating how autonomous yet interconnected *Blue Campuses* could collaborate through exchanges and joint programmes across regions. Lines indicate potential pathways for knowledge, innovation, education and cultural exchange connecting islands and coastal hubs in Europe, Africa, America, and Asia – Pacific. Each node represents a potential *Blue Campus*, forming a living system that links ocean science, conservation, art and entrepreneurship to advance ocean literacy and equitable participation in the blue economy. This AI representation was produced using *ChatGPT* and *Adobe Firefly.*

Valuable examples of scaling interdisciplinary ocean learning globally already exist. For example, IMBeR’s ClimEco Summer Schools have demonstrated how short, intensive interdisciplinary training programmes can connect early career marine scientists across institutions and borders (IMBeR, 2026a), while the Interdisciplinary Marine Early Career Network (IMECaN) (IMBeR, 2026b) shows the power of sustained global networks in building interdisciplinary capacity over time (Palacios-Abrantes et al., 2025). At the school level, the Blue School Global Network, a UNESCO-IOC(2024a) initiative now active in approximately 60 countries, has pioneered interdisciplinary, project-based approaches to ocean literacy for school-aged children (UNESCO-IOC, 2024b; Almeida et al., 2025). The *Blue Campus* ecosystem builds upon these models while extending their reach in two important ways: by targeting youth and communities currently excluded from formal marine science pathways, and by embedding learning within permanent physical hubs that closely connect educational spaces to active business, policy and community activities, anchor local identity and enable global exchange.

Partnerships beyond Europe will be essential to establish this global network. Organisations such as the International Union for Conservation of Nature (IUCN), United Nations (UN), Nippon Foundation-Partnership for Observation of the Global Ocean (NF-POGO) and the Western Indian Ocean Marine Science Association (WIOMSA) represent natural strategic partners for advancing knowledge sharing enabled by the *Blue Campus* model, and we hope others will embrace the opportunity and make it their own. Furthermore, we envision exchanges of early-career and established scientists, facilitating the transfer of best practices, leadership approaches and creative solutions to marine challenges across diverse contexts. By emphasising low-cost and open-source tools, such exchanges could ensure that participants are able to apply newly acquired skills upon return to their home institutions, helping to reduce disparities between the priorities of developed regions and the needs of communities in developing countries. This approach aligns with international commitments to equitable and inclusive capacity development, representing a critical step towards a fair and globally sustainable blue economy.

To transform these exchanges into a sustainable global network, a number of enabling conditions are essential. Funding for initiatives like the *Blue Skills Academy* and *Blue Campuses* is acknowledged as one of the most substantial challenges, particularly given that interdisciplinary programmes consistently face lower success rates in traditional competitive funding streams (Bromham et al., 2016; Blythe and Cvitanovic, 2020). A diversified funding model is therefore essential, combining structural government support, philanthropy and self-sustaining revenue streams such as paid training modules, campus residencies for visiting researchers and artists, tourism spending at the ‘Exploraquarium’, commercial spaces for startups and hospitality facilities. This mixed model reduces dependency on any single funding source and builds long-term financial resilience. In Madeira, funding for the *Blue Skills Academy* has, to date, come from a private philanthropic source, with interest expressed for expanding the programme from another foundation. The concept has also been formally recognised within the institutional future strategic planning of ARDITI (Agência Regional para o Desenvolvimento da Investigação Tecnologia e Inovação), the Madeiran government agency overseeing the initiative, reflecting an emerging commitment to its long-term development (ARDITI, 2026). From the beginning, it has been immensely valuable that the programme’s supporters both appreciate and encourage a multi-stakeholder funding approach, such that the initiative is not solely dependent on one entity. This funding model is not unprecedented, with examples including international book and science festivals, science museums, zoos and aquariums (Bultitude et al., 2011; Woodward, 2012; Miranda et al., 2023) that have long drawn on philanthropy, government support and commercial sales for programmes that combine educational, community-focused, touristic and entertainment elements. Although such funding is not trivial to secure and maintain, many initiatives and institutions have found long-term success through this model. Beyond foundation-level financing, recent growth in investment in blue economy sectors, especially within venture capital (European Commission, 2026), presents additional opportunities for ecosystem-level financing: location-based training, talent pooling and ready multi-stakeholder collaborations make the *Blue Campus* model well-positioned for identifying resilient blue investment opportunities (Vincx et al., 2018; Goswami et al., 2026). Such an ecosystem may more readily attract venture capital to coastal communities with a *Blue Campus*, thereby aiding local economic growth and reinforcing regional incentives to maintain the initiative.

On governance, we envision the establishment of a dedicated foundation with diverse founding members drawn from research, education, government, municipalities, civil society and the private sector, ensuring that no single institution dominates and that the *Blue Campus* remains anchored in its community while remaining open to global partnerships. The foundation would need to have an independent Board with decision-making power, comprising its founding members and beholden to clear governing principles that ensure long-term alignment to the *Campus* mission and an on-going service to the community. In the near term, MARE-Madeira is coordinating the development of the *Blue Skills Academy* and the broader *Blue Campus* concept. As the model matures and the foundation is established, day-to-day coordination would transition to a dedicated operational team or director that is accountable to the Board and responsible for managing partnerships, programming and daily activities; this model allows for freedom in operations while ensuring oversight and a balance of power from an independent, community-represented Board. Additional safeguards to ensure the *Campus* remains community-focused and community-run may include contractual agreements with the *Campus*’ commercial ventures such as minimum guaranteed housing arrangements for visiting researchers and artists or the management of the *Exploraquarium* by *Academy* students. Without such safeguards, there is a risk that commercial interests could, over time, overshadow the *Campus*’s core mission of equity, youth engagement and community participation. Embedding mission into the foundation’s governing principles, alongside an independent Board’s regular review of operational alignment, leadership decisions and commercial partnerships against these values, would help mitigate this risk. Strategic institutional partnerships with international actors and programmes will also ensure the sharing of best practice across communities and related initiatives, including those across research, conservation and education. Together, these elements will support the transition of the *Blue Campus* from an isolated idea to a scalable, trusted and sustainable global network.

Just as crucial as financing and governance is the embedding of inclusion and equality from the start. This means ensuring gender balance in participation and leadership, enabling access for underrepresented groups and small institutions, and designing activities that are socially, financially and physically accessible to all. In island and coastal regions such as Madeira, this also includes creating opportunities for young people from socially and economically disadvantaged backgrounds, who are often closest to the ocean yet furthest from ocean-related education and employment pathways. Building mechanisms that enable their full participation, even if it changes the trajectory of only a single young person at a time, is central to the purpose of the *Blue Skills Academy* and to the social value of a wider *Blue Campus* network. Such diversity is essential for genuine co-creation and aligns with the UN SDG commitment to leave no one behind in building a sustainable blue economy (Lee et al., 2020; Harden-Davies et al., 2022). Ultimately, the success of blue sustainability will not only be measured by expanded infrastructure, new technologies or international partnerships, but also by whether it creates real opportunities for individuals, especially those who have historically been excluded from ocean-related pathways.

Although the *Blue Campus* remains in a conceptual stage, early exchanges already demonstrate its potential to act as a bridge between regions, fostering reciprocal learning and shared innovation. These exchanges also illustrate the power of networked *Blue Campuses* to counter the isolation often felt on islands and in small coastal communities by providing access to mentors, international role models and a vibrant dialogue that inspires innovation and leadership. Crucially, by encouraging locally adapted learning rather than replication, the *Blue Campus* network can empower a new generation of ocean professionals who are globally connected, locally engaged and collectively committed to sustainable blue futures.

In recent years, support for ocean sustainability has directed valuable investment towards research, conservation and innovative business ventures. Yet, building a truly sustainable blue future requires a different kind of investment: in people, especially young people. If even a single student, including those from a socially or economically disadvantaged background, gains the skills and confidence to pursue an ocean-positive career, the *Blue Skills Academy* and the wider *Blue Campus* network will already have begun to fulfil its purpose: demonstrating that meaningful change can begin with a single opportunity.

## Data Availability

Data availability is not applicable to this article as no new data were created or analysed in this study.

## Long descriptions

### Figure 1. Long description

The diagram consists of five stacked horizontal rectangles, each numbered 1 through 5 on the left side. A blue triangular outline sits atop the stack like a roof.

* 1. ACCESSIBILITY AND INCLUSION. Opens new career pathways to diverse and vulnerable youth.

* 2. OPEN-SOURCE INNOVATION. Propagates use of low-cost, open-source tools.

* 3. TRANSDISCIPLINARITY. Co-designed by all interested stakeholders.

* 4. YOUTH EMPOWERMENT. Fostering agency and leadership.

* 5. COMMUNITY ENGAGEMENT. Strengthening local connectivity.

Navigate back to Figure 1..

### Figure 2. Long description

Panel A is a line drawing with numbered labels. 1 is a harbor with a pier and lighthouse. 2 is a cluster of multi-story buildings for the Blue Skills Academy. 3 is the Exploraquarium situated higher on the hill. 4 is a cafe with an outdoor pool area on the shoreline. 5 indicates startup offices. 6 marks conservation and government offices. 7 is a residence hall. 8 is a recreational center at the far right base of the hill.

Panel B is a photorealistic A I interpretation of the same layout. It shows white buildings with blue solar-paneled roofs nestled against a rocky cliffside. A white research vessel is docked in the harbor. The cafe at the water’s edge features a bright blue glass facade. Pedestrian paths connect the various levels of the campus, and the surrounding landscape is arid with sparse green vegetation.

Navigate back to Figure 2..

## References

[r1] Almeida M, Laranjeiro D, Costa R, Lillebø AI and Vieira H (2025) Blue economy concepts are lagging behind in ocean literacy. Frontiers in Marine Science 12, 1648508. 10.3389/fmars.2025.1648508.

[r2] Andrews EJ, Harper S, Cashion T, Palacios-Abrantes J, Blythe J, Daly J, Eger S, Hoover C, Talloni-Alvarez N, Teh L, Bennett N, Epstein G, Knott C, Newell SL and Whitney CK (2020) Supporting early career researchers: Insights from interdisciplinary marine scientists. ICES Journal of Marine Science 77(2), 476–485. 10.1093/icesjms/fsz247.

[r3] ARDITI (2026) Relatório Anual 2025. Agência Regional para o Desenvolvimento da Investigação Tecnologia e Inovação. Madeira, Portugal. Available at https://www.arditi.pt/pt/noticias/relatorio-anual-2025.html (accessed June 2026).

[r4] Back to Blue Initiative (2023) The Great Disconnect: Young People and Ocean Literacy. The Economist Impact. Available at https://backtoblueinitiative.com (accessed June 2026).

[r41] BBNJ Treaty (2023) Agreement under the United Nations Convention on the Law of the Sea on the Conservation and Sustainable Use of Marine Biological Diversity of Areas beyond National Jurisdiction. United Nations, New York. https://www.un.org/bbnjagreement/en.

[r5] Bennett NJ, Cisneros-Montemayor AM, Blythe J, Silver JJ, Singh G, Andrews N, Calò A, Christie P, di Franco A, Finkbeiner EM, Gelcich S, Guidetti P, Harper S, Hotte N, Kittinger JN, le P, Lister J, López de la Lama R, McKinley E, Scholtens J, Solås AM, Sowman M, Talloni-Álvarez N, Teh LCL, Voyer M and Sumaila UR (2019) Towards a sustainable and equitable blue economy. Nature Sustainability 2(11), 991–993. 10.1038/s41893-019-0404-1.

[r6] Blythe J and Cvitanovic C (2020) Five organizational features that enable successful interdisciplinary marine research. Frontiers in Marine Science 7, 539111. 10.3389/fmars.2020.539111.

[r7] Bromham L, Dinnage R and Hua X (2016) Interdisciplinary research has consistently lower funding success. Nature 534(7609), 684–687. 10.1038/nature18315. 27357795

[r8] Bultitude K, McDonald D and Custead S (2011) The rise and rise of science festivals: An international review of organised events to celebrate science. International Journal of Science Education, Part B 1(2), 165–188. 10.1080/21548455.2011.588851.

[r9] Castro N, Ramalhosa P, Jiménez J, Costa JL, Gestoso I and Canning-Clode J (2020) Exploring marine invasions connectivity in a NE Atlantic Island through the lens of historical maritime traffic patterns. Science of the Total Environment 37, 101333. 10.1016/j.rsma.2020.101333.

[r10] Cisneros-Montemayor AM, Moreno-Báez M, Reygondeau G, Cheung WWL, Crosman KM, González-Espinosa PC, Lam VWY, Oyinlola MA, Singh GG, Swartz W, Zheng CW and Ota Y (2021) Enabling conditions for an equitable and sustainable blue economy. Nature 591(7850), 396–401. 10.1038/s41586-021-03327-3. 33731948

[r11] Coyle D (2010) The Talent Code. London: Arrow Books.

[r12] Esson D, Acenas I, Alves F, Bender S, Czaplinski T, De La Torre PR, Dierking J, Dubbeld J, Freitas JR, Gouveia M, Howe B, Kumar V, Kurinchi-Vendhan R, Maggipinto B, Monteiro J, Motsenbocker B, Nunes P, Oswald JN, Pestana J, Phillips B, Pinheiro M, Prager A, Prager Urbina P, Prieler M, Radeta M, Redaelli L, Rost T, Schinault M, Shannon M, Semedo SV, Singh H, Stenico F, Strauss S, Tomasi B, Varini F, Verkhovskaya A, Vieira AD, Weihl L, Zhang MM and Canning-Clode J (2025) The Impossible Things Workshop 2025: A Review. MARE-Madeira. Available at https://twilighted.eu/resources/ (accessed June 2026).

[r13] Esson D, Dierking J, Phillips B, Oswald JN, Zhang MM, Kurinchi-Vendhan R, Pinheiro M, Acenas I, Rost T, Freitas R, Prieler M, Dubbeld J, Radeta M, Semedo S, Singh H, Kumar V, Varini F, De La Torre P, Motsenbocker B, Vieira D, Pestana J, Nunes P, Alves F, Monteiro JG and Canning-Clode J (2026) Accelerating deep-sea research through accessible technologies. Trends Open. 10.1016/j.treopn.2026.04.008.

[r14] European Commission (2025) EU Blue Economy Report 2025. Publications Office of the European Union, Luxembourg. Available at https://oceans-and-fisheries.ec.europa.eu (accessed June 2026).

[r15] European Commission: Directorate-General for Maritime Affairs and Fisheries (2026) The next wave of blue growth – Investor report 2026. Publications Office of the European Union. Available at https://data.europa.eu/doi/10.2771/1823987 (accessed June 2026).

[r16] García ACA (2017) New ports of the New World: Angra, Funchal, Port Royal and Bridgetown. The International Journal of Maritime History 29(1), 155–174. 10.1177/0843871416677952.

[r17] Goswami S, Goswami BK and Srivastava RK (2026) Strategic human resource development for a sustainable blue economy: Developing ocean-smart talent. In Gupta VP, Reyed RM and Haghi AK (eds.), The Blue Economy and Environmental Sustainability: Advancing Global Governance, Innovation, and Finance for a Resilient Future. World Sustainability Series. Cham: Springer Nature Switzerland. 10.1007/978-3-032-13638-1_24.

[r18] Harden-Davies H, Ota Y, Fardin F, Schutter M, Singh G and Sey A (2022) Listen to ocean voices for an equitable ocean decade. Marine Technology Society Journal 56(3), 16–17. 10.4031/MTSJ.56.3.7.

[r19] Hoareau K (2025) Knowledge, power and the blue economy: Breaking down barriers. Marine Policy 182, 106883. 10.1016/j.marpol.2025.106883.

[r20] IMBeR (2026a) ClimEco Summer Schools. Integrated Marine Biosphere Research. Available at https://imber.info/events/summer-schools/ (accessed June 2026).

[r21] IMBeR (2026b) IMECaN — Interdisciplinary Marine Early Career Network. Integrated Marine Biosphere Research. Available at https://imber.info/imecan-interdisciplinary-marine-early-career-network/ (accessed June 2026).

[r22] Kadykalo AN, Affinito F, Hamilton J, Galang EINE, Pentz B, Cornejo-Denman L, Ferguson D, Howard SC, Kobluk HM, Owen KC, Zhang Y, Obroh OG, Roughan BL and Wells EE (2026) Co-envisioning an academia that fully embraces and supports early career researchers in interdisciplinary social-ecological research. Ecology and Society 31(1), 17. 10.5751/ES-16694-310117.

[r23] Kelly R, Mackay M, Nash KL, Cvitanovic C, Allison EH, Armitage D, Bonn A, Cooke SJ, Frusher S, Fulton EA, Halpern BS, Lopes PFM, Milner-Gulland EJ, Peck MA, Pecl GT, Stephenson RL and Werner F (2019) Ten tips for developing interdisciplinary socio-ecological researchers. Socio-Ecological Practice Research 1(2), 149–161. 10.1007/s42532-019-00018-2.

[r24] Kenney M (2000) Understanding Silicon Valley: The Anatomy of an Entrepreneurial Region. Stanford, CA: Stanford University Press. 10.1515/9781503618381.

[r25] Lee K-H, Noh J and Khim JS (2020) The blue economy and the United Nations’ sustainable development goals: Challenges and opportunities. Environment International 137, 105528. 10.1016/j.envint.2020.105528. 32014791

[r26] Loroño-Leturiondo M and Olazabal M (2025) The underexplored potential of the arts in environmental social sciences. Environmental Science & Policy 172, 104224. 10.1016/j.envsci.2025.104224.

[r27] MARE-Madeira (2025) 2024 Impact Report. Madeira, Portugal. Available at https://mare-madeira.pt/impact/ (accessed June 2026).

[r28] Martínez-Vázquez RM, Milán-García J and de Pablo Valenciano J (2021) Challenges of the blue economy: Evidence and research trends. Environmental Sciences Europe 33(1), 61. 10.1186/s12302-021-00502-1.

[r29] McKinley E, Burdon D and Shellock RJ (2023) The evolution of ocean literacy: A new framework for the United Nations ocean decade and beyond. Marine Pollution Bulletin 186, 114467. 10.1016/j.marpolbul.2022.114467. 36516497

[r30] Miranda R, Escribano N, Casas M, Pino-del-Carpio A and Villarroya A (2023) The role of zoos and aquariums in a changing world. Annual Review of Animal Biosciences 11(1), 287–306. 10.1146/annurev-animal-050622-104306. 36266060

[r31] Palacios-Abrantes J, Dias B, Gianelli I, Strand M, Zuo F, Hong G and Qin K (2025) The role of the interdisciplinary marine early career network (IMECaN) in supporting capacity building, ocean literacy, and collaborative leadership of early career researchers. Limnology and Oceanography Bulletin 34(2), 53–55. 10.1002/lob.10704.

[r32] Radeta M, Monteiro JG, Pestana J, Vieira D, Abreu P, Silva R, Schäfer S, Ramalhosa P, Martos AL, Loureiro B, Queiroz N, Seabra R, Lima FP and Canning-Clode J (2025) MARS: Programmable multipurpose auto-release system for aquatic observations. Limnology and Oceanography: Methods 23, 376–388.

[r33] Saxenian A (1996) Regional Advantage: Culture and Competition in Silicon Valley and Route 128. Cambridge, MA: Harvard University Press.

[r34] UNESCO-IOC (2024a) Ocean Literacy Guide. United Nations Educational, Scientific and Cultural Organization, Paris. Available at https://oceanliteracy.unesco.org (accessed June 2026).

[r35] UNESCO-IOC (2024b) Blue School Global Network. United Nations Educational, Scientific and Cultural Organization — Intergovernmental Oceanographic Commission. Available at https://oceanliteracy.unesco.org/projects/blue-school-global-network (accessed 15 June 2026).

[r36] UNESCO-IOC (2026) Projects. Ocean Literacy Portal, Intergovernmental Oceanographic Commission. Available at https://oceanliteracy.unesco.org/projects (accessed 15 June 2026).

[r37] Vincx M, Antia A, Deprez T, Fiksen Ø, Koski M, Mackenzie B, McGrane P, O’Carroll C, Olsen J, Menezes Pinheiro L, Ribera d’Alcalà M and Roullet G (2018) Training the 21st century marine professional: A new vision for marine graduate education and training programmes in Europe. *Future Science Brief No. 2.* European Marine Board, Ostend. Available at https://www.marineboard.eu/sites/marineboard.eu/files/public/publication/EMB_FSB2_2018_Web_v1.pdf (accessed June 2026).

[r38] Wenhai L, Cusack C, Baker M, Tao W, Mingbao C, Paige K, Xiaofan Z, Levin L, Escobar E, Amon D, Yue Y, Reitz A, Neves AAS, O’Rourke E, Mannarini G, Pearlman J, Tinker J, Horsburgh KJ, Lehodey P, Pouliquen S, Dale T, Peng Z and Yufeng Y (2019) Successful blue economy examples with an emphasis on international perspectives. Frontiers in Marine Science 6, 261. 10.3389/fmars.2019.00261.

[r39] Woodward S (2012) Funding museum agendas: Challenges and opportunities. Managing Leisure 17(1), 14–28. 10.1080/13606719.2011.638202.

[r40] World Bank and United Nations Department of Economic and Social Affairs (2017) The Potential of the Blue Economy: Increasing Long-Term Benefits of the Sustainable Use of Marine Resources for Small Island Developing States and Coastal Least Developed Countries. Washington, DC: World Bank.

