# Supplementary material for: The missing link in the blue economy: Empowering the next generation through blue skills and ocean literacy
Source: Camb Prism Coast Futur. 2026 Jul 7;4:e25. doi: 10.1017/cft.2026.10042 (PMC13748885; doi:10.1017/cft.2026.10042)
Supplement: Peer review history

# Reviewer report: Review: The missing link in the blue economy: Empowering the next generation through blue skills and ocean literacy — R0/PR2

**DOI:** 10.1017/cft.2026.10042.pr2

**Authors:** Michelle Voyer

**Affiliations:** University of Wollongong

The proposed Blue Skills Academy and Blue Campuses presented in this perspective piece provide an inspiring and forward‑looking vision, and I really appreciate the clear commitment to interdisciplinarity, systems thinking, and equity. Positioning these principles at the centre of the proposal gives the piece a refreshing sense of ambition and moral purpose. The emphasis on breaking down disciplinary silos and embedding inclusive practice throughout is particularly compelling and aligns well with contemporary thinking about the future of the blue economy.

That said, the overall tone leans toward the conceptual, and several practical considerations remain underdeveloped. The concept would benefit from a more concrete articulation of how the model is intended to function in real‑world conditions. Key operational questions, such as funding sources, governance, administration, and long‑term sustainability, are left largely unanswered. Who would be responsible for coordinating such a model? What mechanisms would ensure continuity beyond initial enthusiasm or pilot‑phase investment? Without clarity on these structural elements, the proposal risks appearing aspirational rather than actionable.

I think the piece could also do with a little clearer articulation of the nature, scale and scope of the purported gap in “blue skills” training. It is not yet clear whether the issue is a genuine absence of training opportunities, a lack of shared understanding of what constitutes blue skills, or simply a fragmented and siloed landscape where existing programs are poorly connected. Distinguishing between these possibilities is essential, as each implies a different problem, and therefore a different solution. It would also help identify how and where the proposed Academy/campuses would ‘fit’ within the broader training and education landscape (eg connections with established higher education bodies etc, certification standards for skills training etc).

Finally, the proposal would benefit from a clearer explanation of how this model differs from established structures such as maritime clusters. Many regions already operate cluster‑based approaches that bring together industry, education, research, and government. If the proposed model is intended to go beyond these, the piece should articulate the added value: Is it the systems‑thinking orientation? The equity‑driven design? The interdisciplinary approach?

Overall, the perspective piece offers a compelling vision with exciting ideas and strong conceptual foundations. Additional detail and evidence as outlined above would help to strengthen this vision into a more concrete, pragmatic and actionable pathway towards fulfilling this vision.

# Reviewer report: Review: The missing link in the blue economy: Empowering the next generation through blue skills and ocean literacy — R0/PR3

**DOI:** 10.1017/cft.2026.10042.pr3

**Authors:** Jessica Blythe

**Affiliations:** Environmental Sustainability Research Centre, Brock University, Canada

Dear authors,

Thank you for the opportunity to review your perspective paper, The Missing Link in the Blue Economy: Empowering the Next Generation Through Blue Skills and Ocean Literacy. This manuscript addresses a timely and important gap, namely the need to invest in “blue skills,” understood here as interdisciplinary (and potentially transdisciplinary) training for early-career marine professionals. The concept of a Blue Skills Academy is compelling, the five core principles are relevant and well aligned with current challenges in the marine and blue economy sectors, and the paper is clearly written and accessible to a broad audience.

That being said, there are several opportunities to further strengthen the manuscript.

First, the contribution of the paper could be more clearly situated within the existing body of scholarship on interdisciplinary training and capacity building in marine and ocean sciences. While the need for such training is well articulated, engaging more explicitly with prior frameworks, programs, or empirical studies on interdisciplinary or transdisciplinary marine education would help clarify how this perspective builds on, complements, or advances existing approaches. Doing so would not only strengthen the academic grounding of the paper but also help readers better understand what is novel about the proposed Blue Skills Academy and its core principles. Literature such as:

https://link.springer.com/article/10.1007/s42532-019-00018-2

https://doi.org/10.1093/icesjms/fsz247

https://doi.org/10.1002/lob.10704

Second, the manuscript would benefit from more explicitly acknowledging existing interdisciplinary marine centres/schools/institutions, and then describing how the Blue Skills Academy builds on, advances, or is unique from these models.

For instance, in relation to Vision: Part 1: Blueprint for a Blue Campus, there are numerous well-established and successful examples of interdisciplinary marine schools and centres that could serve as useful points of comparison. Some relevant examples include:

Marine Institute (https://www.mi.mun.ca/aboutus/) - As a campus of Memorial University of Newfoundland, the Fisheries and Marine Institute is Canada’s most comprehensive centre for education, training, applied research and industrial support for the ocean industries (note, while focused on many technical marine programs, they also engage in community-based training and other interdisciplinary perspectives).

Centre for Marine Socioecology (https://marinesocioecology.org/) - CMS provides, develops and integrates multi-disciplinary research to better understand and manage Australia’s oceans.

Moreover, in relation to Vision: Part 2 – Scaling through exchange, there are wonderful interdisciplinary marine schools that specifically focus on scaling interdisciplinary marine science globally. For example,

IMBeR’s (https://imber.info/) ClimEco Summer Schools (https://imber.info/events/summer-schools/) & IMECaN (https://imber.info/imecan-interdisciplinary-marine-early-career-network/)

And the Blue School Global Network: a toolkit (which is admittedly geared towards school-aged children).

While it might be out of scope (and is not necessary for the manuscript), it might be useful to run a gap analysis of the existing interdisciplinary marine institutions to help justify the unique contribution/niche of the Blue Skills Academy.

Third, there are some significant challenges for supporting initiatives like these – such a funding - that are well established in the literature, and it would be useful to engage with them by acknowledging the challenges and suggesting actions to address/overcome them. For example, these papers identify that funding for interdisciplinary marine science can be particularly challenging:

https://doi.org/10.5751/ES-16694-310117

https://www.frontiersin.org/journals/marine-science/articles/10.3389/fmars.2020.539111/full

Finally, several minor comments.

You use the term ‘interdisciplinary’ throughout to describe the integration of various academic disciplines, but it is not defined. Since it’s a core concept in your perspective, I suggest adding a definition and citation. The definitions in Figure 1 of this article could work: https://link.springer.com/article/10.1007/s42532-019-00018-2

P.3 In Box 1 – I suggest adding equitable to the first definition (‘Sustainable and equitable blue economy’ rather than just ‘Sustainable blue economy’). You rightfully emphasize the importance of inclusion and accessibility throughout (and already cite Bennett et al. 2019 which advocates for a sustainable and equitable blue economy).

p. 4 Providing an example of how each of the five core principles will be operationalized would strengthen this section.

# Editor report: Recommendation: The missing link in the blue economy: Empowering the next generation through blue skills and ocean literacy — R0/PR4

**DOI:** 10.1017/cft.2026.10042.pr4

**Affiliations:** Institute for the Oceans and Fisheries, Institute for the Oceans and Fisheries, Canada

Dear Dr. Canning-Clode and co-authors, 

Thank you for submitting your manuscript to Cambridge Prisms: Coastal Futures. I am pleased to inform you that you have received two substantive, excellent and thoughtful reviews, and I am grateful to both reviewers for the care and constructive engagement they have brought to this manuscript. Both reviewers found the manuscript timely, engaging, and aligned with current discussions about the future of the blue economy and ocean literacy. At the same time, both reviewers suggest that the manuscript would benefit from further development in several areas to strengthen its scholarly grounding and practical relevance. I therefore invite you to submit a major revision (given the breadth of revisions requested across framing, literature engagement, and operational grounding) that addresses all the points raised by the reviewers before the manuscript can advance toward publication.

I would like to underline three areas in particular. 

First, in a context of limited resources, (new) initiatives benefit from demonstrating clear synergies with, rather than duplication of, existing efforts.The manuscript would therefore benefit from acknowledging relevant precedents and articulating more explicitly what is distinctive about the proposed model. In doing so, and in line with reviewer 2, please provide examples of relevant approaches and existing programmes and/or scholarly frameworks that utilise these approaches and articulate clearly how your proposal builds upon or fills gaps not addressed by these. While a comprehensive gap analysis is out of scope (as indicated by reviewer 2), demonstrating an understanding of 1) existing approaches and 2) existing institutions that make key contributions of the kinds you highlight is in my mind necessary to situate your contribution within existing scholarship and practice. I would also ask you to reflect explicitly on whether the vision you describe necessarily requires an entirely new programme, or whether it could strengthen or connect existing ones. 

Second, and building on the resource sustainability dimension, both reviewers note that aspects related to governance, funding, sustainability, and administration are underdeveloped. While you do not need to provide a full operational plan, the manuscript would benefit from acknowledging the known challenges (see Reviewer 2 suggestions) and outlining enabling conditions more concretely. Reviewer 1’s questions are helpful in integrating specific points in your revised manuscript in this regard.

Third, as suggested by Reviewer 2 “Interdisciplinary” is used throughout but never defined; please add a definition and citation. I would also encourage you to engage explicitly with transdisciplinarity, which goes beyond integrating disciplines to include collaboration and co-creation of knowledge and solutions with diverse stakeholders. Transdisciplinarity strikes me as a unique opportunity to: a) more accurately capture the nature of the collaboration you are proposing and the knowledge processes at the heart of the Academy; b) strengthen the equity and inclusion argument as transdisciplinary approaches support democratising knowledge production and legitimising non-expert and indigenous ways of knowing; c) differentiate the Academy from other/more conventional interdisciplinary marine education programmes.

Please accompany your revision with a point-by-point response to both reviewers and to the comments above.

I look forward to receiving your revised manuscript.

With kind regards,

Colette Wabnitz [handling editor]

# Editor report: Decision: The missing link in the blue economy: Empowering the next generation through blue skills and ocean literacy — R0/PR5

**DOI:** 10.1017/cft.2026.10042.pr5

**Affiliations:** Geography, Cambridge University, United Kingdom of Great Britain and Northern Ireland

No accompanying comment.

# Editor report: Recommendation: The missing link in the blue economy: Empowering the next generation through blue skills and ocean literacy — R1/PR7

**DOI:** 10.1017/cft.2026.10042.pr7

**Affiliations:** Institute for the Oceans and Fisheries, Institute for the Oceans and Fisheries, Canada

Dear João Canning-Clode and co-authors.

Thank you for revising the manuscript in response to the reviewers’ comments and for outlining how the revisions were addressed. I appreciate the effort the authors have invested in strengthening the manuscript. I have only a few minor comments remaining before I would be comfortable recommending the manuscript for publication.

Graphical abstract: In my mind, the graphical abstract does not yet appear to effectively communicate the central message of the paper. As currently presented, it is difficult to interpret without accompanying text, and the icons alone do not provide sufficient information for readers to readily understand the main concepts or take-home messages. Consider revising the graphical abstract to more clearly convey the purpose and key elements of the proposed model.

References: The reference list still remains relatively limited given the breadth of work that has been undertaken in areas such as ocean literacy, capacity development, interdisciplinary and transdisciplinary training, and blue economy education. While I appreciate the authors’ efforts to incorporate the specific literature suggested by the reviewers, I had hoped to see a somewhat broader engagement with the wider body of relevant scholarship beyond these additions. Expanding the literature base would further strengthen the manuscript’s scholarly grounding and help situate the proposed model more clearly within existing research and practice.

Main text

Vision Part 1: Thank you for the additions situating the Blue Campus concept within existing initiatives. This is helpful. My suggestion, building on that from reviewer 1, would be to articulate which specific gaps existing models do not fully address and how the proposed model seeks to fill those gaps – this could be done by highlighting a couple of examples.

Vision Part 2: The additional discussion is appreciated, particularly the clearer positioning of the Blue Campus ecosystem in relation to existing programmes and networks. The expanded discussion on enabling conditions is also helpful. However, the statement that “light governance that prioritises transparency, inclusion and trust, and equitable access made possible through open-source tools and low-cost participation would further support this vision” remains somewhat vague. A little more specificity regarding what this governance arrangement might look like in practice would strengthen the argument.

Building on the points above, while many of Reviewer 1’s concerns have been addressed, one key question remains only partially answered: “Who would be responsible for coordinating such a model?” I agree with the authors’ response to the HE that a full

operational blueprint is beyond the scope of this Perspective. However, providing a clearer indication of / some dimensions that would underpin how coordination might be structured would help make the vision feel more tangible and achievable rather than primarily aspirational.

On a related note, The proposed diversified funding model, combining government support, philanthropy, and self-sustaining revenue streams, is sensible. While hospitality facilities, commercial spaces, tourism-related activities, and other revenue-generating components may contribute to financial sustainability, it would be useful to acknowledge the need for safeguards that ensure these activities remain aligned with, and supportive of, the core mission. Without such safeguards, there could be a not-insignificant risk that commercial interests could gradually overshadow the initiative’s primary objectives, particularly its commitments to equity, youth engagement, and community participation.

Moreover, given the current funding landscape, it would be helpful to provide some indication of whether there is evidence that such a model is attainable in practice. While the manuscript provides useful examples of preliminary partnerships, sponsorships, and pilot activities that demonstrate early momentum behind the concept, it remains less clear whether there is evidence of possible longer-term financial support and broader supportive engagement. For example, it would be helpful to provide some indication of whether discussions, partnerships, expressions of interest, or funding efforts are already underway that suggest a pathway from the current pilot initiatives toward the larger campus model.

Acknowledgments: Finally, I would encourage the authors to acknowledge the valuable contributions of the reviewers. Their comments have significantly strengthened the manuscript and helped improve its scholarly grounding and practical relevance.

Many thanks and kind regards,

Colette

# Editor report: Decision: The missing link in the blue economy: Empowering the next generation through blue skills and ocean literacy — R1/PR8

**DOI:** 10.1017/cft.2026.10042.pr8

**Affiliations:** Geography, Cambridge University, United Kingdom of Great Britain and Northern Ireland

No accompanying comment.

# Editor report: Recommendation: The missing link in the blue economy: Empowering the next generation through blue skills and ocean literacy — R2/PR10

**DOI:** 10.1017/cft.2026.10042.pr10

**Affiliations:** Institute for the Oceans and Fisheries, Institute for the Oceans and Fisheries, Canada

No accompanying comment.

# Editor report: Decision: The missing link in the blue economy: Empowering the next generation through blue skills and ocean literacy — R2/PR11

**DOI:** 10.1017/cft.2026.10042.pr11

**Affiliations:** Geography, Cambridge University, United Kingdom of Great Britain and Northern Ireland

No accompanying comment.
